# Nanograin size effects on the strength of biphase nanolayered composites

**DOI:** 10.1038/s41598-017-10064-z

**Published:** 2017-09-12

**Authors:** Sixie Huang, Irene J. Beyerlein, Caizhi Zhou

**Affiliations:** 1 Department of Materials Science and Engineering, Missouri University of Science and Technology, Rolla, MO 65409 USA; 2 Department of Mechanical Engineering, Materials Department, University of California at Santa Barbara, Santa Barbara, 93106 USA

## Abstract

In this work, we employ atomic-scale simulations to uncover the interface-driven deformation mechanisms in biphase nanolayered composites. Two internal boundaries persist in these materials, the interlayer crystalline boundaries and intralayer biphase interfaces, and both have nanoscale dimensions. These internal surfaces are known to control the activation and motion of dislocations, and despite the fact that most of these materials bear both types of interfaces. From our calculations, we find that the first defect event, signifying yield, is controlled by the intralayer spacing (grain size, *d*), and not the intralayer biphase spacing (layer thickness, *h*). The interplay of two internal sizes leads to a very broad transition region from grain boundary sliding dominated flow, where the material is weak and insensitive to changes in *h*, to grain boundary dislocation emission and glide dominated flow, where the material is strong and sensitive to changes in *h*. Such a rich set of states and size effects are not seen in idealized materials with one of these internal surfaces removed. These findings provide some insight into how changes in *h* and *d* resulting from different synthesis processes can affect the strength of nanolayered materials.

## Introduction

Two-phase nanolayered (NL) metallic composites are one of the few nanostructured materials that uniquely exhibit a multitude of attractive structural and functional properties, ranging from high strength, ductility, hardness, radiation resistance, to thermal stability^1–4^. Very recently, advanced manufacturing methods have been employed to successfully make NL materials in bulk, that is, in sizes suitable for large structures. Scaling up in this way enables exploitation of their exceptional suite of properties in a much broader range of applications than thought possible^5, 6^.

NL composites are comprised of alternating layers of two metal phases, which individually are less than 100 nm. Usually one metallic nanocrystal spans an individual layer thickness *h*, joining from one bimetal interface to the other. Many studies on the strength of these materials find that decreasing *h* can strengthen the material, particularly when *h* lies in the nanoscale range, from 100 nm to 10 nm. It is believed that the nanoscale dimensions affect the selection of deformation mechanisms, such as dislocation glide and sliding along the interfaces or grain boundaries, that determine material strength, differently than in coarser dimensions and this nanoscale alteration grows as *h* decreases. The dislocation core itself has nanoscale grain size dimensions, and thus, the movement of just one dislocation within a crystal, for instance, can have a noticeable impact on the strength of the entire NL composite. However, how *h* affects dislocation motion (including production and annihilation) needs to be better understood in order to identify the relation between *h* and the strength of NL composites.

A large number of studies over the past decade, involving *in-situ* TEM, diffraction, atomic-scale modeling, and dislocation theory, have been devoted to understanding how interfaces affect dislocation motion in strained nanolayered materials^7–15^. Many theories and MD simulations have shown that interfaces can act as sources, sinks, barriers, and/or storage sites for dislocations and deformation twins^9–12^. Li *et al*. revealed that interfaces in bimetal NL composites can provide the high diffusivity and vacancy concentration for promoting dislocation climb at room temperature^16^. In some experimental studies, a limit value of the critical layer thickness, *h*_c_, has been reported, below which strength no longer increases but plateaus or drops. Using dislocation theory, the strongest value of *h*_c_ has been postulated to occur at the crossover from confined layer slip to slip transfer across the bi-phase boundaries^17^. Yet, whether or not a limit *h*_c_ is found, the reported sensitivity of NL strength to *h* can vary among studies on similar NL materials^18^ Much of the variability can be attributed to different choices of strength measures, either yield or peak strength in tension or compression, or indentation hardness, or to processing-induced variations in the microstructure, such as texture or the in-plane sizes of the grains *d* within the nanocrystalline (NC) layers^19^. It, therefore, becomes apparent that understanding role that interfaces and their densities and spacing play in affecting dislocation motion, and therefore strength, would help in rationalizing these results.

One prominent nanostructural feature that is missing in most studies is the nanocrystalline grain structure of the individual layers. Two length scales, therefore, should be used to describe NC NL composites: *h* the mean distance between interfaces (IFs) and the grain size *d*, the mean in-plane distance between adjacent GBs. Collecting knowledge gained from studies in either NC materials or NL nanocomposites indicates that both GBs and IFs would greatly affect the dynamics and kinetics of dislocations in strained materials. To date, not many studies involving calculations or theories have been carried out to understand the coupled effect of GBs and IFs on the deformation of NL composites. A majority of the MD work that connects grain boundary affected dislocation motion, nanograin size, and strength pertain to single-phase nanocrystalline (NC) metals^19–22^. Most NL modeling studies treat the layers as single crystalline and not as NC^7–10^. Recently, Zhu *et al*.^14, 15^ investigated size effects in nanolayerd polycrystalline metallic multilayers by MD simulations and found that the micro-plasticity deformation can be dominated by several possible dislocation mechanisms. While both length scales *h* and *d* could be feasibly altered in manufacturing, the values for *h* and *d* needed to achieve the highest yield or flow strength are not known. A key question then arises: which length scale, *h* or *d*, dominates and controls the peak strength or the onset of softening? Is it plausible to believe that the finest length scale, the one that is the closest in length scale to the dislocations, would be the one that controls strength of the material? To date, there are no calculations or theories that consider the coupled roles in deformation to confirm or deny this or any notion regarding the coupled effect of grain boundaries and interfaces on dislocation nucleation and motion.

In this article, we use MD simulation to explore the coupled effects of *h* and *d* on the yield and flow strength of NC NL composites. We apply the study to a Cu/Nb nanolaminate with a nanostructure of one made by physical vapor deposition (PVD). We show that the strongest microstructural length scales do not correspond to the one with the finest dimension in both *h* and *d*. The grain size *d* affects the sensitivity of strength to reductions in layer thickness *h* increasing as *d* decreases. Once the material is deforming plastically, the flow stress is governed by the relative contributions of grain boundary-driven dislocation emission and grain boundary sliding. Although both are related to the grain boundaries, both *d* and *h* are found to govern the relative contributions of these two mechanisms. Analysis of the relative contributions of different grain boundary mechanisms (dislocation emission and subsequent slip vs. grain boundary sliding) explain that decreasing *d* can result in higher contributions of grain boundary sliding, a weaker composite, and reduced strength improvements with decreasing *h*. These results reveal that understanding the strength of nanostructured materials involves considering both *d* and *h*.

## Material and Nanostructure

**Figure 1:**
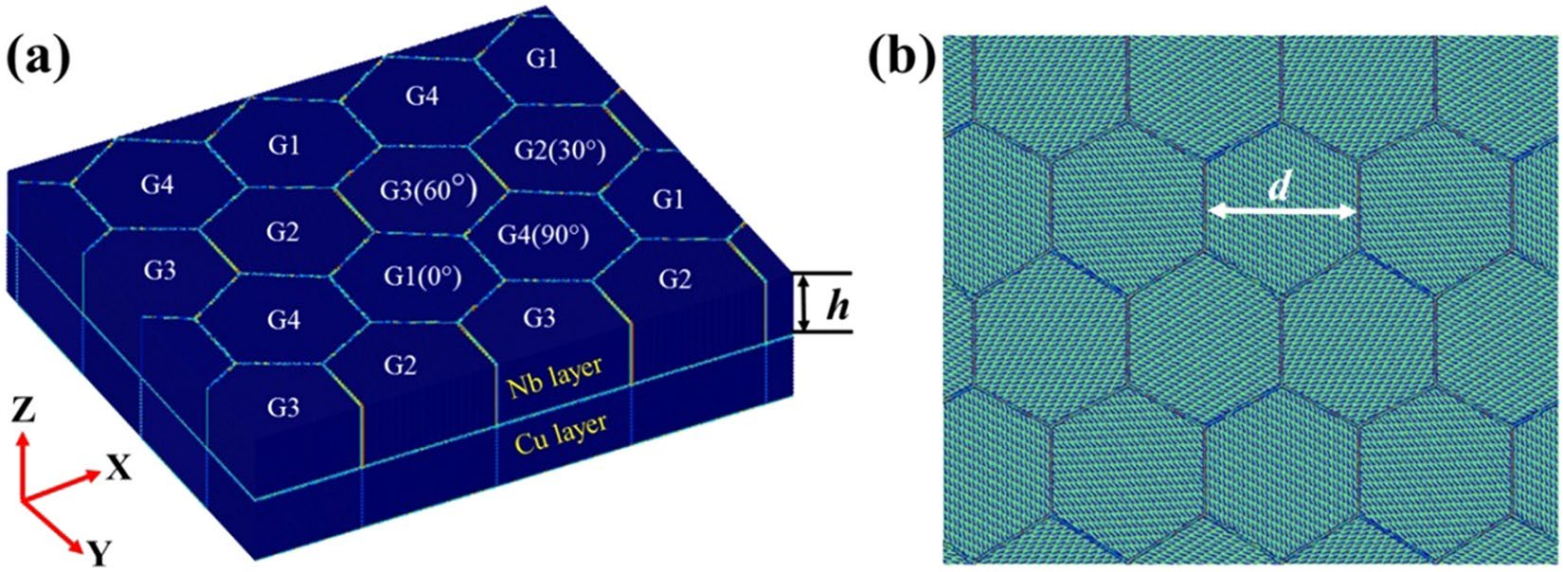
(**a**) Atomic scale configurations of nanograined Cu/Nb multilayers, (**b**) relaxed interface pattern in different grains (top view), colored according to the centro symmetry parameter^33^. (The directions for three axis are $[1,\bar{1},0]\,$Cu||$[\bar{1},1,1]\,$Nb in X axis, $[1,1,\bar{2}]\,$Cu||$[1,\bar{1},2]\,$Nb in Y axis and $[1,1,1]\,$Cu||$[1,1,0]\,$Nb in Z axis).

MD simulations of Cu/Nb multilayers were performed with the Large-scale Atomic/Molecular Massively Parallel simulator (LAMMPS) code^23^. Figure 1(a) shows the simulation cell for the polycrystalline (PX) NL Cu/Nb composites. Periodic boundary conditions have been applied to all three directions of this cell. The forces between Cu-Cu, Nb-Nb and Cu-Nb atoms were calculated by the interatomic potential^2, 24, 25^ based on the Embedded Atom Method (EAM)^26^. This potential has been used previously in several studies on defect nucleation, formation, interactions, and propagation and replicates key defect properties, such as the energy of the stacking faults created by gliding partial dislocations^8, 10–12^.

Each layer is composed of four grains, and four Cu/Nb grain pairs connect across the interface. These hexagonal columnar grains were created by the Voronoi tessellation method^27^. To match the microstructure common for Cu/Nb composites synthesized via physical vapor deposition, the crystallographic orientation between each pair of Cu/Nb grains was made to follow the Kurdjumov-Sachs (KS) orientation relationship^28^, meaning the directions in Cu and Nb for three axes are such that [1, 1, 0]Cu||[1, 1, 1]Nb are aligned in the X-axis, [1, 1, 2]Cu||[1, 1, 2]Nb in the Y-axis and [1, 1, 1]Cu||[1, 1, 0]Nb in the Z-axis. To create the nanograined sample, we fixed one pair of grains as the initial crystallographic orientation and rotated the other three pairs of Cu/Nb grains by 30°, 60° and 90° degree along the Z-axis. Consequently, grain boundaries were created in each layer while maintaining the KS orientation relationship for each pair of Cu/Nb grains.

With all the above nanostructural aspects fixed, we then proceeded to create NL NC composites with different combinations of *d* and *h*. Many different grain sizes, *d*, were used: 2.5, 5, 10 nm, 20 nm and 40 nm and as well as values of *h*, the layer thickness: 2.5 nm to 15 nm. The largest number of atoms in this model is about 15,000,000. In all cases of *h* and *d*, the grains had the same hexagonal shape and hence the same number of connecting triple junctions *per grain*.

Before loading, all NL composites were relaxed under the conditions associated with an isobaric isothermal ensemble (NPT^27^, constant pressure and temperature) at zero pressure and 1 K for 300 ps via a Nose-Hoover temperature thermostat and pressure barostat^29, 30^. This relaxation step allows the atoms to readjust their coordinates and settle into a lower energy state. Figure 1(b) shows the relaxed interface pattern for each pair of Cu/Nb grains according to the centro-symmetry parameter. These patterns are consistent with those reported in earlier work but for single crystalline (SX) Cu/Nb multilayers^31, 32^.

After relaxation, the NC NL composites are subjected to uniaxial tension parallel to the X-axis in Fig. 1(a) such that Cu/Nb interfacial sliding would not be encouraged. In all cases to follow, we applied a constant strain rate of 5 × 10^8^ s^−1^. The time interval for each simulation step was 1 fs.

## Results and Discussion

### Nanostructure effects on stress-strain response

**Figure 2:**
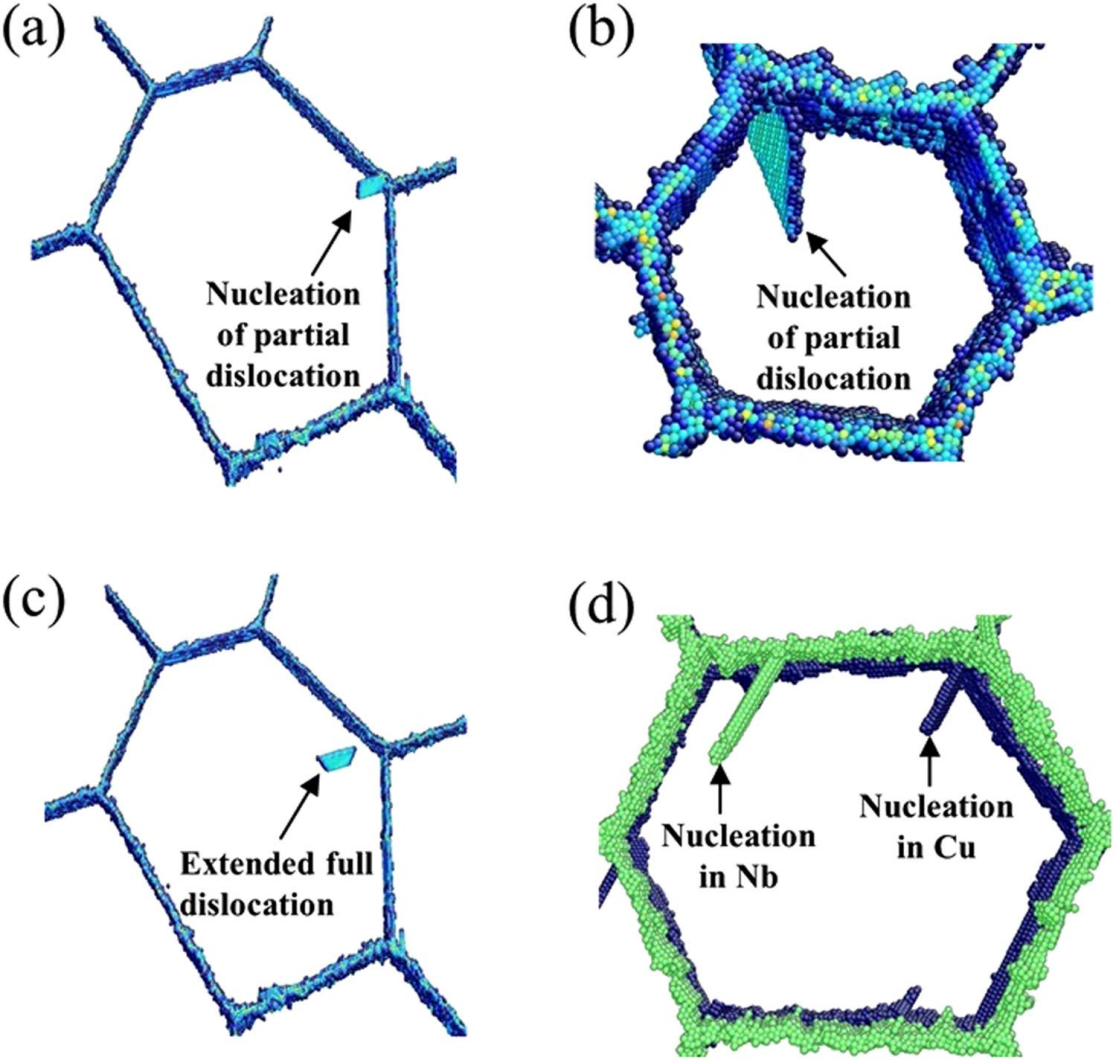
(**a**) The nucleation of partial dislocation from GB in Cu layer (*d* = 40 nm), (**b**) the nucleation of partial dislocation from GB in Cu layer (*d* = 10 nm), (**c**) extended full dislocation glide in Cu layer (*d* = 40 nm), and (**d**) the nucleation of partial dislocations from GB in both Cu and Nb layer (*d* = 20 nm), the interface atoms has been set as transparent.

Upon loading the NC NL composites, leading Shockley partial dislocations initially emit from the grain boundary triple junctions, where the grain boundaries and interfaces meet, rather than from the bimetal interfaces. An example of this grain boundary dislocation emission (GBE) event is shown in Fig. 2(a) and (b). The partials can be followed by a trailing partial either shortly afterwards, such that a full dislocation glides across the grain as showed in Fig. 2(c), or later in time after a stacking fault has already been formed across the grain by the leading Shockley partial. Secondly, we observe that the GBE occurs in both Cu and Nb as shown in Fig. 2(d).

These results have a few important distinctions from single crystalline (SX) NL composites. Such MD simulations have been reported earlier in this Cu/Nb KS system^7–10^ but since some finer details in model set up and boundary conditions may be different, we carried out analogous simulations single crystalline (SX) NL composites and they are reported in the supplemental material section. Results presented there are consistent with those made previously. Firstly, under the same loading state, leading Shockley partial dislocations would initially emit from the bimetal interfaces in SX NL composites (see Figure S2 in supplemental material). Secondly, dislocations emit into both phases in the NC NL composites, unlike in the SX NL where dislocations first emit into Cu and later into Nb. In NC NL composites, the preferred location for dislocation nucleation is the junction between interfaces and grain boundaries, since a large local stress concentrations tend to develop at those sites.

**Figure 3:**
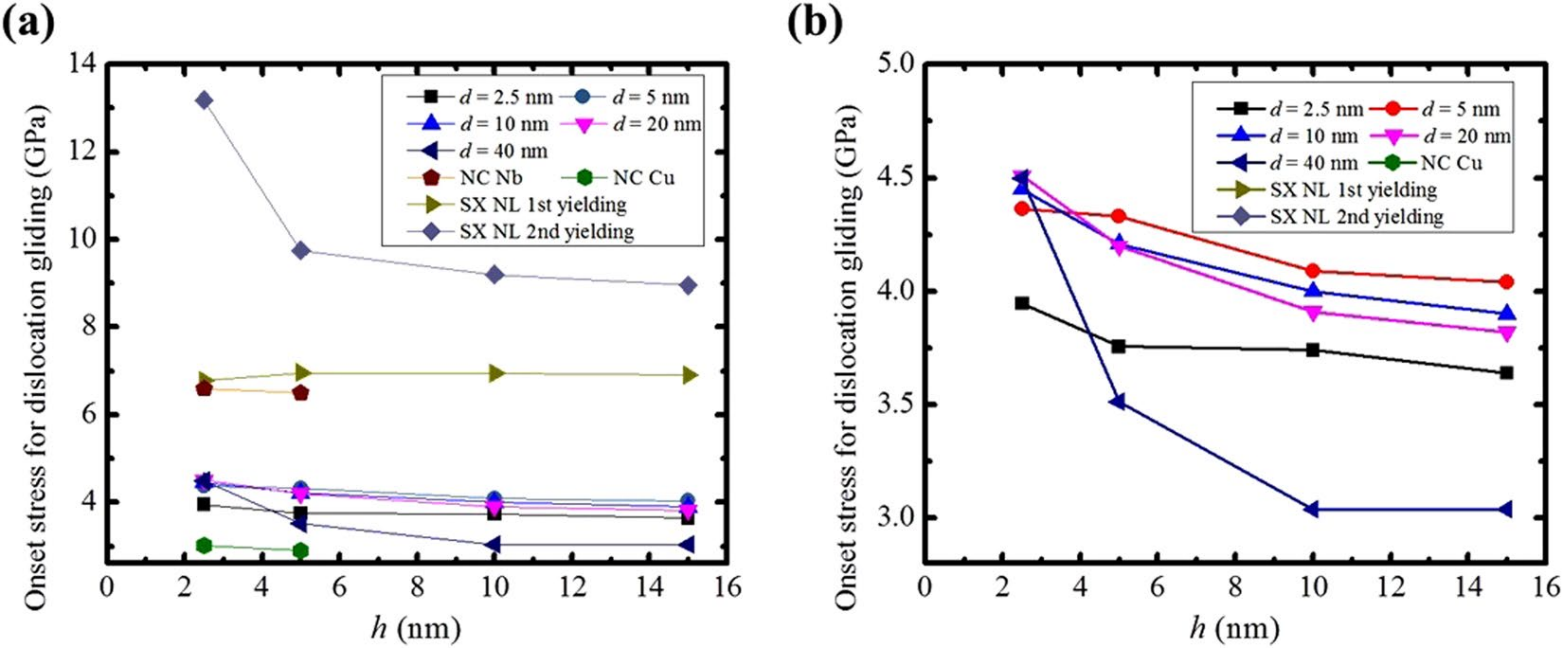
Plots of the onset stress for dislocation glide as a function of layer thickness, *h*, (**a**) for NC NL, SX NL, NC Nb and NC Cu, (**b**) only for NC NL.

Figure 3 show the onset stress for dislocation glide, while Fig. 3(b) only consider the cases of NC NL composites and Fig. 3(a) include the cases of SX NL and NC Cu. The stress-strain curve for all cases were shown in supplemental material. The effect of having nanocrystalline layers with grain boundaries is to weaken the NL relative to the ideal SX NL composite with the same *h*. This result implies that by virtue of how dislocations are nucleated that SX NL provides a practical upper bound to the strength of NC NL materials with the same *h*. This same viewpoint would, in turn, also suggest that nanocrystalline Cu (nc) with similar, equiaxed *h* = *d* grain sizes would be even weaker, providing an apparent lower bound. These analogous simulations were also carried out and checked against literature values (see supplement material Figs 1 and 2). Some strength values are reported in Fig. 3(a). As expected, the strength of the NC NL composites lies between those of the SX NL and NC Cu for the same range of *h* and *d*. Relative to the NC Cu with the same grain width *d* and height *h*, the Cu/Nb interfaces in the NC NL substantially strengthen the material.

Thus we find that the introduction of grain boundaries cause the NC NL composites to become weaker relative to the SX NL for two reasons: the dislocations emit more easily from grain boundaries than the Cu/Nb interfaces and the GBE enables simultaneous plastic deformation in both Cu and Nb.

### Mechanisms governing yield stress

Figure 3(b) shows the variation in NC NL yield stress with *h* and *d*. Generally with respect to the yield stress, we observe the much anticipated scaling: smaller is stronger–as *d* and *h* both decrease, the yield stress increases. However, there are two exceptions. First, independent of *h*, a critical value *d*_s_ exists where *d*_s_ = 2.5 nm, the yield drops. Second, for the finest, *h* = 2.5 nm, the yield stress is highest at *d* = 20 nm and decreases with reductions in *d* from 10 nm to 2.5 nm. Interestingly, the NC Cu also exhibits the same trend; the yield stress is the highest for *d* = 20 nm and decreases with reductions in *d* from 10 nm to 2.5 nm. See Figure S1. In prior NC Cu simulations studies^34, 35^, this transition has been associated with a transition from slip-dominated deformation above the peak value *d* ~ 10–20 nm to grain boundary sliding-dominated deformation below. It would hint that even in NC NL composites, the grain boundaries or their spacings (grain sizes) in the nanocrystalline layers are driving the type of yield event.

**Figure 4:**
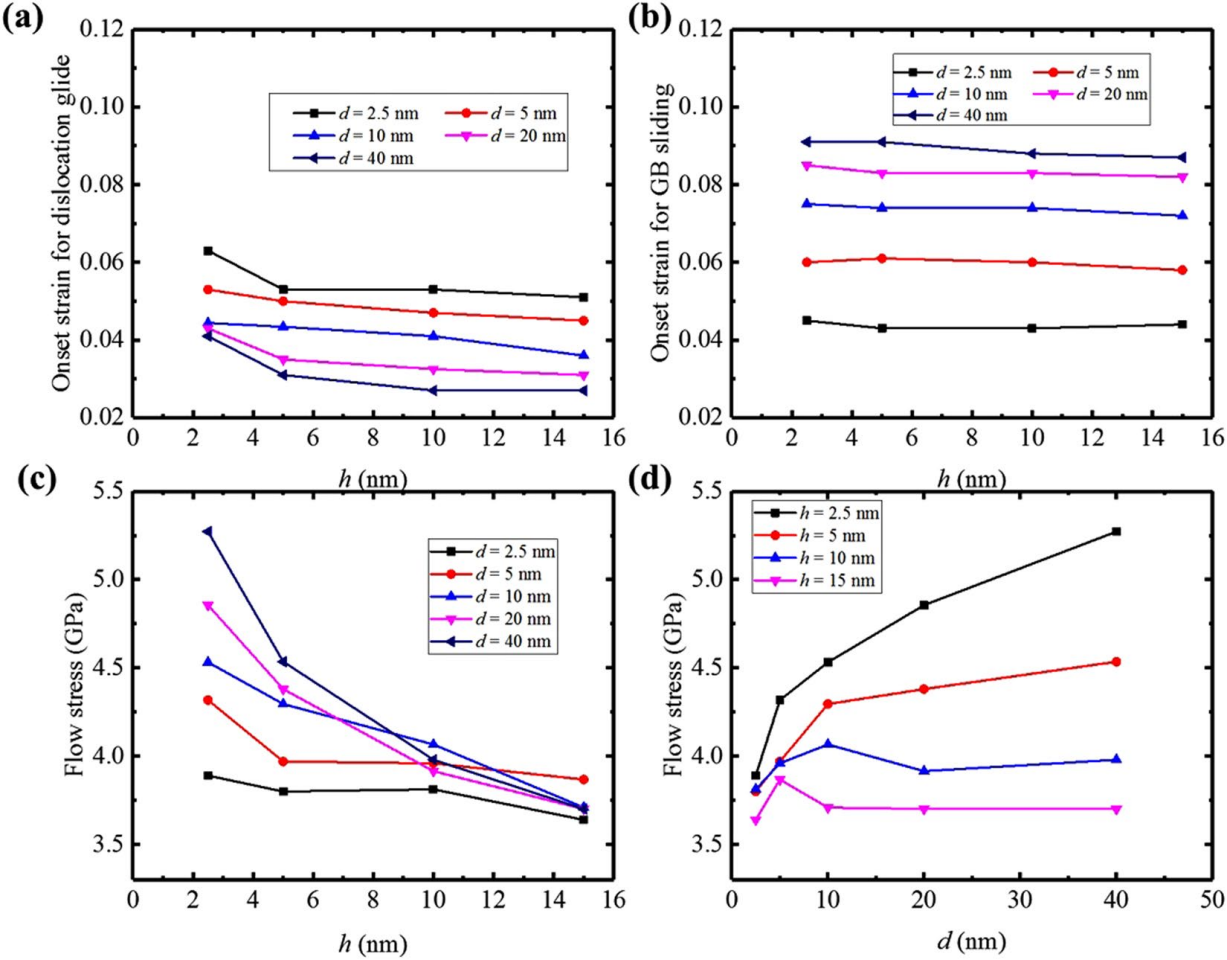
The strain for onset by (**a**) grain boundary dislocation emission and (**b**) GB sliding versus layer thickness. (**c**) The flow stress (average over 7~12% strain) vs layer thickness, *h* (**d**) the flow stress vs grain size *d*.

To determine more specifically the grain-boundary-driven mechanisms responsible for yield, we employ the atomic-shift analysis to determine the strain at which dislocation emission and grain boundary sliding first occur in each nanolaminate. Figure 4 shows how the onset strain for GBE and GBS vary with *h* and *d*. For all *d* above *d* = 2.5 nm, the onset strain for dislocation emission is less than the onset strain for grain boundary sliding. Thus, emission of a dislocation from the grain boundaries marks the end of the linear regime and hence determines the true yield stress. The grain size *d*_s_ = 2.5 nm signifies a critical point when *d* is small enough that the onset strain for GBS (~0.045%) is lower than that for GBE and the onset of GBS is responsible for yield of the NC NL composite. For most values of *h, d*_s_ is also the value of *d* for which the yield strength of the material reduces rather than increases and the peak yield strength is realized for *d* = 5 nm, just above *d*_s_. This behavior was not seen in the NC Cu cases, wherein the yield strength increased proportionally with increase in *d*.

The value of *d* at which *peak yield* is reached appears to be well correlated with the critical value of grain size *d*_s_ marking a transition from GBE to GBS. However, for the finest NC NL *h* = 2.5 nm, the peak yield is reached at *d* = 20 nm, well above *d*_s_ (see Fig. 3(b)). For cases in which GBE governs composite yield (*d* > *d*_s_), a further distinction between partial and full dislocation emission can be made. *Partial GBE* involves emission of a leading partial, which traverses the grain and forms a stacking fault across the grain, and emission of the trailing partial at a later time in strain. *Full GBE*, on the other hand, means that after the leading partial emits from the grain boundary, the trailing partial emits soon after, such that a full dislocation traverses the grain and no grain-scale stacking fault forms. As described earlier, the partial and full dislocations, particularly at the onset of yield, can be identified. Normally, larger grain can provide longer mean free path for the leading partial that leaves enough time for the trailing partials to emit from the GB and for a full GBE. Employing the atomic-shift analysis technique at the onset strains for *d* > *d*_s_, we find that for small *d*, *d* < 10 nm, partial GBE defines yield but for large *d* > 20 nm, full GBE marks the end of linear elastic deformation. The grain size *d* = 20 nm is a transition region when partial GBE occurs for larger *h* and full GBE for smaller *h*. Thus, in these NC NL composites, the yield strength can be sensitive to whether the first yield event is a partial or full GBE. Higher nucleation stresses are associated with full GBE.

### Mechanisms governing flow stress

From Fig. 4, we observe that in none of the NL nanostructures tested, does either GBS or GBE act alone throughout deformation. Rather it is observed that dislocation emission (whether partial or full) defines yield, with the exception of *d*_s_ = 2.5 nm, and GBS starts after dislocation emission. As *d* increases further beyond *d*_s_, the more GBS is postponed and the more of the plastic strain is carried by dislocation glide. GBS, in these cases, are part of determining the flow stress after yield, but not yield. Likewise, for *d* = 2.5 nm, GBS may control the yield point, but GBE occurs shortly thereafter with more straining. We, therefore, can expect that the mechanisms governing the flow stress after yield would be different from those responsible for yield. Consequently the dependencies of flow stress on *h* and *d* would not necessarily follow those of the yield stress.

Specifically from the NC NL results in Fig. 4, over the stress range of 7–12%, both GBE and GBS have initiated and the NC NL material is flowing with contributions from both mechanisms. Figure 4(c) and (d) analyzes the variation of an average flow stress over the strain range of 7–12% with *h*, the conventional way to assess the strength of nanoscale NL. We considered minor adjustments to this strain range, only to find that they do not alter the trends reported here. It is observed in Fig. 4(c) and (d) that the NC NL flow stress increases as *h* decreases. Generally NL strengthening with smaller *h* in the nanoscale regime is often seen experimentally^17, 28, 36, 37^. With respect to *h*, smaller leads to a higher flow stress. It is, however, a significant finding in Fig. 4(c) and (d) that the size scaling in *h* depends on *d*, weakening as *d* decreases. This result implies that to best exploit layer thickness *h* reductions for increasing strength (i.e., flow stress), the grain size *d* should be as large as possible.

Another important signature of the coupled effects of *h* and *d* is the crossing of the curves in Fig. 4(c). To elucidate it, we plot in Fig. 4(d), the same stress data for NC NL composites of fixed *h* with variation in *d*. For the larger *h* = 15 nm and 10 nm NC NL composites, a critical grain size *d*_c_ can be identified at which the composites achieves peak strength, which is 5 nm and 10 nm, respectively. For the finer *h* NC NL composites, *h* = 2.5 nm and 5 nm, the material weakens as *d* decreases. Evidently *d*_c_ is larger than 40 nm, the largest grain size tested here. The interesting finding is that in NC NL composites, a critical *d*_c_ exists and it depends on *h*, appearing to increase as *h* increases. This size scaling does not resemble the scaling in *d* for the NC Cu with no interfaces or the scaling in *h* for the SX NL composites with no grain boundaries, but follow the general trend of the scaling for flow stresses in NC Nb (see Figure S1).

**Figure 5:**
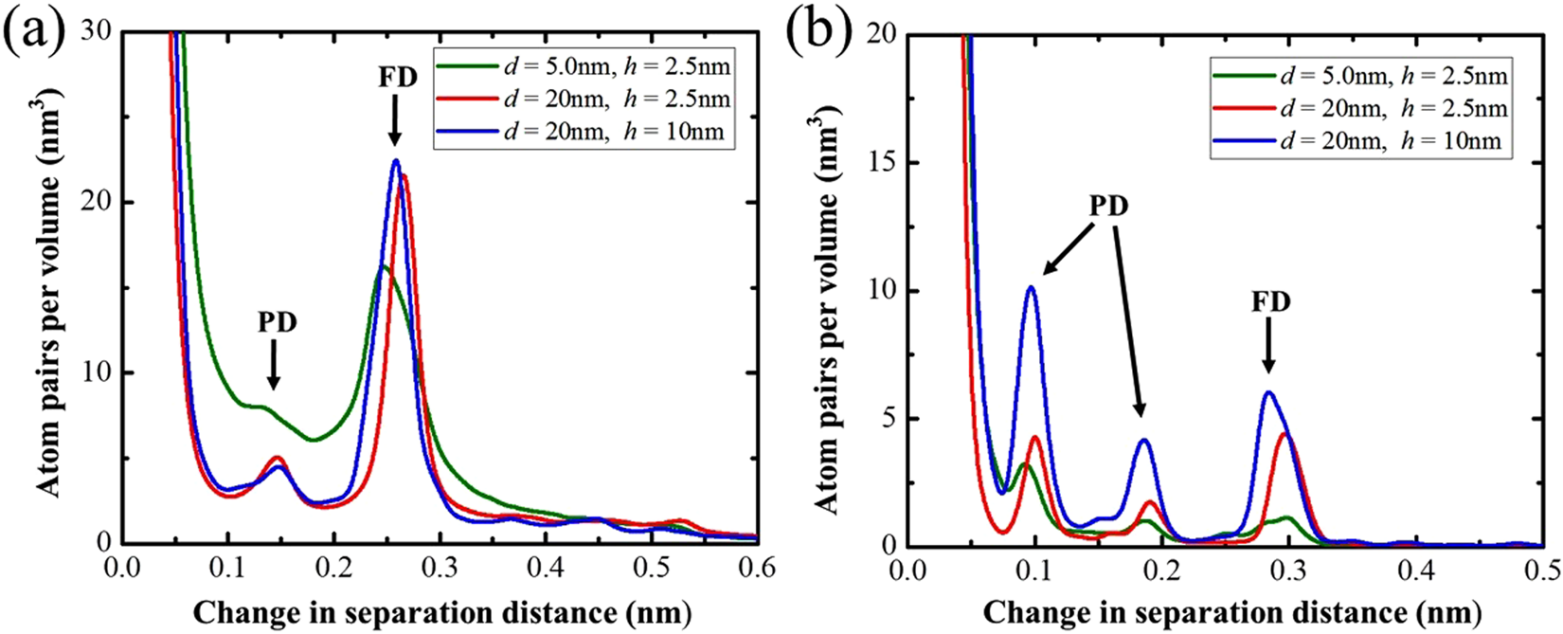
Histogram of the changes in the separation distance of initially nearest neighbor atoms after 10% strain in Cu layer (**a**) and Nb layer (**b**).

To understand how these size effects happen, we first calculate the atomic shifts in the material at 10% strain. The frequency plots for the atomic shifts for a few composites are shown in Fig. 5. Two cases (*h* = 2.5 and *d* = 5 and 20 nm) lie in the softening regime e.g., *d* < *d*_c_ (*h*), and the other one (*h* = 10 nm and *d* = 20 nm) in the hardening region, *d* > *d*_c_ (*h*). Again, we see evidence of dislocation glide activity in both regimes. For the cases shown, we see that there is more dislocations gliding in Cu than Nb, both partial and full dislocations are gliding in Cu and Nb, and most of the dislocations are full dislocations in Cu while most of them are partial dislocations in Nb. Clearly, the amount of dislocation activity is linked strongly to the finest of the microstructural length scales, with less dislocation activity for finer *h* and *d*. However, dislocation glide contributes to carrying the strain whether the material strengthens or weakens with reduction in microstructure scales. Thus, there is not a clear abrupt transition in mechanisms that determines *d*_c_.

**Figure 6:**
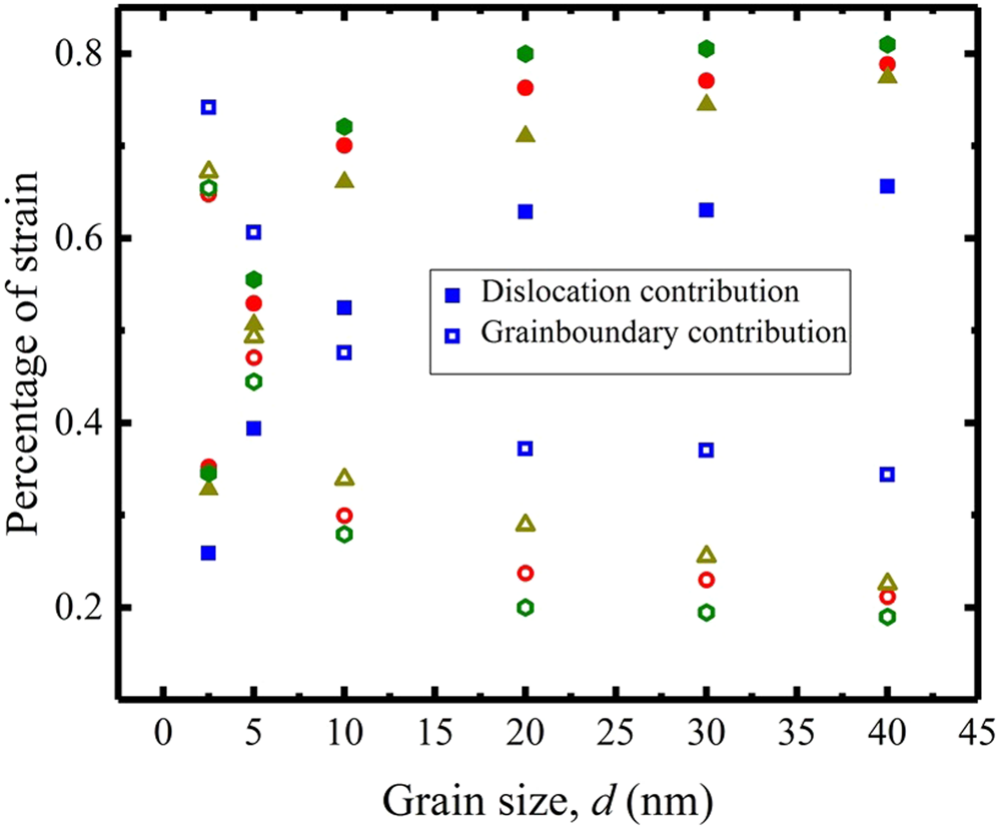
Comparison of the contributions in plastic strain from dislocation gliding (open symbols), and grain boundary sliding (solid symbols) at 10% total strain. (Squares for *h* = 2.5 nm, triangles for *h* = 5.0 nm, circles for *h* = 10 nm and diamonds for *h* = 15 nm).

From the atomic-shift analysis, the relative amounts of GBE and GBS can be assessed at any given strain. Figure 6 shows their relative contributions as a function of *h* and *d*. We notice that in all cases, the strain throughout the deformation test is accommodated by a combination of GBE and GBS. The relative amounts of GBS increase as *d* deceases. For *d* = 5 nm, their contributions are nearly equivalent (~50%). For *d* = 2.5 nm, GB sliding dominates strain accommodation (>60%). This analysis makes clear that for the range of *d* and *h* studied, GBE and GBS contribute to strain accommodation within the material. However, their relative amounts are sensitive to the two microstructural length scales *h* and *d*. The value of dc in flow stress corresponds to when the GBS contribution exceeds a threshold value of 25%, regardless of the value of *h*. Thus at the strongest nanoscale microstructural combination, dislocation glide will still carry most of the deformation (~75% or more). Further, at the transition size *d*_c_, GBE defines yield and carries plasticity after yield.

**Figure 7:**
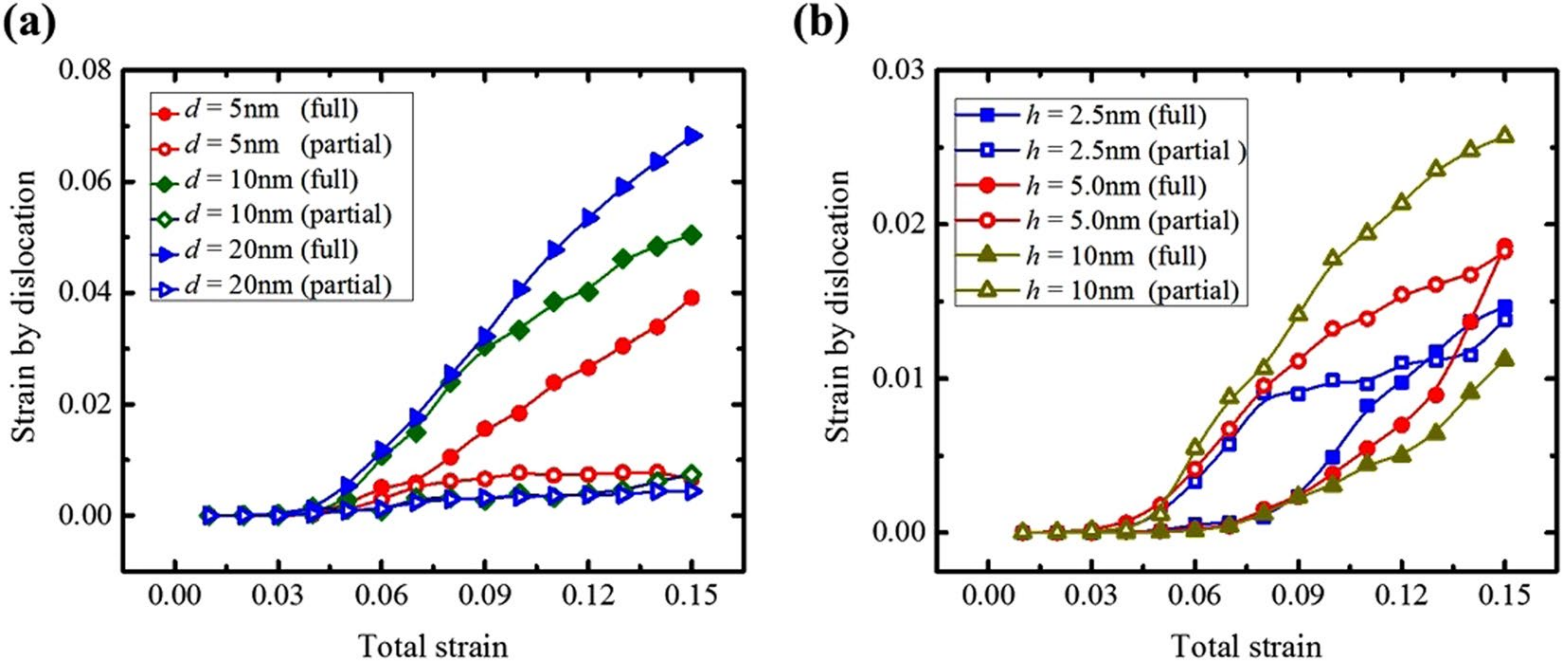
Strain contributions from dislocation slip for different samples in Cu layers: (**a**) h = 2.5 nm with different grain sizes, (**b**) *d* = 2.5 nm with different layer thicknesses.

From the foregoing analysis, we find that in most cases, dislocation glide mediates plastic strain. In such fine nanocrystals, partial slip rather than full slip is generally thought to carry most of the strain. To determine the contribution of partial or full dislocation slip over the entire deformation response we applied atomic shift analysis. In Fig. 7(a), we first show for *h* = 2.5 nm, a very finely layered nanolaminate for a range of grain sizes, 5 nm to 20 nm, wherein the GBE dominates the flow stress. In these cases, the first yield event is SF formation. However, from Fig. 7(a), we see that after more strain, full dislocation glide dominates in all cases. In Fig. 7(b), we analyze the evolution of dislocation activity in cases where GBS dominates (*d* = *d*_s_). We see an interesting correlation between partial slip and GBS, partial glide dominates over the entire straining period. After emission, these dislocations glide across the crystal by threading through the layers. Theoretically, the finer *h*, the more stress required for an individual dislocation to push through ~log(*h*)/*h*. Consequently, the finer *h*, the higher the flow stress. Unlike, the yield stress associated with stress to emit the first dislocations, depends on *h*. This effect can be seen in Fig. 7(b), as *h* decreases, more total applied strain is needed to achieve the same dislocation strain.

**Figure 8:**
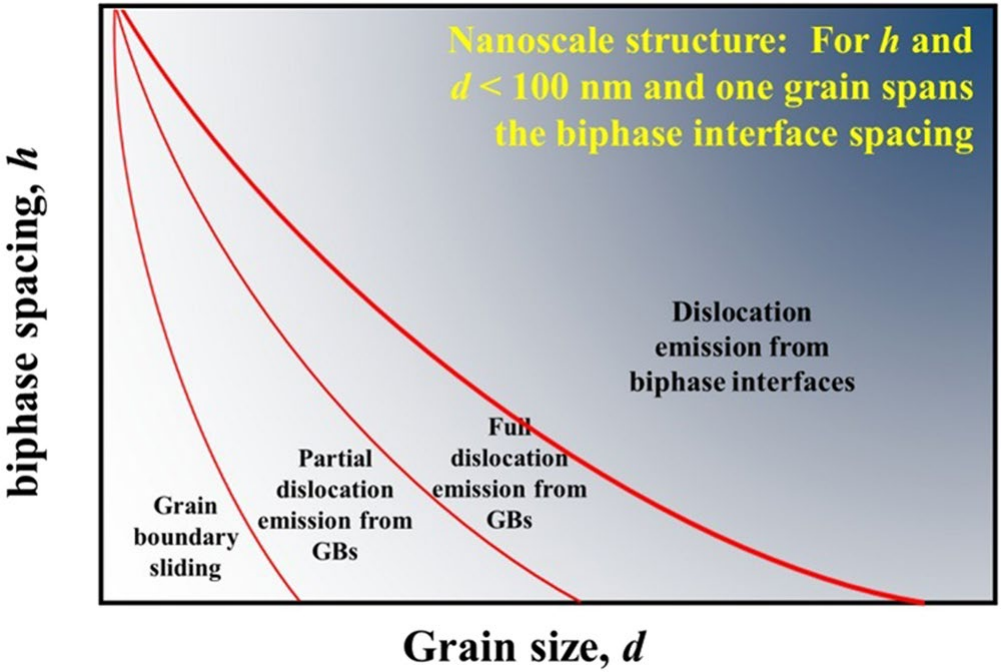
Generalized mechanism map for the first yield event in strained NC NL composites.

The present simulation results on Cu/Nb nanolayers and those from a recent similar study on polycrystalline Cu/Ag nanolayers^14^ help to clarify the size-driven mechanisms that affect the yield stress of NC NL To illustrate this, Fig. 8 maps the regimes for the predominant deformation mechanisms underlying yield on a plot with axes *h* and *d*. This map would apply to nanoscale materials in which only one grain spans the layer thickness *h* and the grains are *d* in width and both *d* and *h* have nanoscale dimensions (<100 nm, such as in Fig. 1). At one end of the map, with large *d*, yield is determined by first emission of a dislocation from the biphase interface. At the other end, with small *d*, yield is determined by grain boundary sliding. In between, as *d* and *h* increase yield is governed by emission of partials and full dislocations from the grain boundaries.

## Conclusions

In summary, we use atomic-scale simulation to investigate microstructural size scaling in the strength of nanocrystalline nanolayered (NC NL) Cu/Nb composites. Scalings in both the intralayer NC grain size *d* and layer thickness *h* were investigated. The calculations reveal strongly coupled *d*-*h* effects. Unlike single crystalline nanolayered composites without grain boundaries, where plasticity is initiated by emission of dislocations from the interfaces into preferably one of the phases, in the NC NL composites, dislocations are emitted from the junctions were grain boundaries and interfaces meet and within both phases. Both phases, thus, participate in yield and plastic flow in NC NL. Further, the grain size *d* controls the yield phenomenon, with the finest of grain sizes *d* ≤ *d*_s_, yielding via intralayer grain boundary sliding (GBS), and the larger grain sizes *d* > *d*_s_, yielding by intralayer grain boundary dislocation emission (GBE). Grain size *d* also governs the relative amounts contributed by GBE and GBS during plastic flow stress after the defining first yield event has occurred. The highest flow stress (strongest) NC NL occurs at a grain size *d*_c_, the grain size below which the percentage contribution of GBS equals or increases greater than 25%. As GBS becomes increasingly hindered as *h* increases, the value of the “strongest size *d*_c_” for the NC NL composite decreases as *h* increases. Last, the intragranular grain boundary spacing *d* also affects the sensitivity of NC NL strength to reductions in *h*. Partial dislocation activity occurs when GBS dominates and the effect of *h* on strength is weak, whereas full dislocation activity prevails when GBS is small (<60%) and the smaller the amounts of GBS, the greater gains in strength with reduction in *h*. The grains should be as large as possible to best reap the strengthening benefits of reductions in layer thickness.

The foregoing results on layer *h* size and intralayer *d* effects in NC NL composites make clear that the average size *d* of the grains in the nanocrystalline layers is a highly influential variable for strength. In most cases, the grain sizes among NL composites of different *h* are not reported or not the same. These findings can help to better interpret *h*-scale effects on measure yield or flow strength.

## Methods

To identify the mechanisms responsible for deformation, we used two procedures. AtomEye^38^ was used to visualize the configuration of atoms in the microstructure. The second one is denoted here as the atomic-shift analysis and is built upon the methods provided by Vo *et al*.^39^ for nanocrystalline fcc metals. This analysis determines the relative amounts of partial dislocation glide, full dislocation glide, and grain-boundary-mediated deformation. In brief, the first step involves identifying the atoms in the grain interiors using the Common neighbor analysis (CNA)^25, 40^. The second step calculates the pair separation (PS) for the atoms in grain interiors. PS is a measure of the relative motion between nearest neighbor pairs of atoms compared to its initial value. From this analysis, we can evaluate the frequency of atomic shifts for the entire system at any given strain level during deformation. Characteristic amounts of shifts in these plots correspond to the Burgers vector of either partial or full dislocations. More details of this method are given in Vo *et al*.^39^. Further, from these atomic shifts, the amount of strain contributed by partial or full dislocation glide can be calculated by summing the strain induced by all atoms displaced by dislocation motion, as follows:

1 $${\varepsilon }^{dis}=\sum _{i}^{N}\frac{A}{V}\times (\bar{l}\cdot {\bar{b}}_{i})\times (\bar{l}\cdot {\bar{n}}_{i})$$

where ${\bar{b}}_{i}$ is the Burgers vector of the dislocation slipping over the atom *i*, ${\bar{n}}_{i}$ is the unit normal of the slip plane for the dislocation slipping over the atom *i*, $\bar{l}$ is the loading direction, $A$ is the unit area of atoms projected on the slip plane, $V$ is the volume of the simulation box, and *N* is the total number of slipped atom.

All other atomic shifts not associated with dislocation glide are attributed to grain boundary deformation, such as grain boundary sliding (GBS) and diffusion. Since the current simulations are carried out a 1 K, it is likely that these atomic shifts can be attributed predominantly to grain boundary sliding (GBS). In this article, the atomic-shift analysis is used to determine the onset strain and the relative contributions of dislocation glide or GBS to accommodating strain at any strain level.
